# Exploration of the Spanish Version of the Attachment Style Questionnaire: A Comparative Study between Spanish, Italian, and Japanese Culture

**DOI:** 10.3390/ejihpe11010010

**Published:** 2021-02-04

**Authors:** Oscar López-de-la-Nieta, Mᵃ Alejandra Koeneke Hoenicka, José Luis Martinez-Rubio, Kazuyuki Shinohara, Gianluca Esposito, Giuseppe Iandolo

**Affiliations:** 1 Department of Psychology, School of Biomedical Sciences, European University of Madrid, Spain. Calle Tajo S/N. (Urb. El Bosque), Villaviciosa de Odón, 28670 Madrid, Spain; oscar.lopezdelanieta@psisemadrid.com (O.L.-d.-l.-N.); mariaalejandra.koeneke@universidadeuropea.es (M.A.K.H.); jluis.martinez@universidadeuropea.es (J.L.M.-R.); 2 Department of Neurobiology & Behavior, School of Biomedical Sciences, Nagasaki University, 1-12-4 Sakamoto, Nagasaki 852-8523, Japan; kazuyuki@nagasaki-u.ac.jp; 3 Social and Affective Neuroscience Lab, Psychology Program-SSS, Nanyang Technological University, Singapore 639818, Singapore; 4 Lee Kong Chian School of Medicine, Nanyang Technological University, Singapore 636921, Singapore; 5 Affiliative Behaviour and Physiology Lab, Department of Psychology and Cognitive Science, University of Trento, 38068 Rovereto, Italy

**Keywords:** attachment, ASQ Spanish, intercultural research

## Abstract

Nowadays, there are several human attachment measures, most in the form of questionnaires that assess adult attachment styles. This study investigates the use of Feeney, Noller, Hanrahan, Sperling and Berman’s five-factors Attachment Style Questionnaire (ASQ, 1994), based on Bartholomew’s four-factors model (1991), and Hazan and Shaver’s three-factors model (1987). Nevertheless, no robust study has explored the ASQ questionnaire in Spanish compared to other cultures, such as Italian and Japanese. Therefore, the linguistic translation of the Spanish version of the ASQ was performed, based on the back-translation methodology. The results indicate that 5-factors ASQ Spanish version explains 43.67% of the variance, similar to the original English-Australian ASQ version. The Italian and Japanese versions explain 49.37% and 52.27% of the variance, respectively. No age correlation for any ASQ factors in the Japanese sample was found; meanwhile, the Spanish and Italian cultures showed a positive correlation with age and “Confidence” and negative correlation with age and “Relationships as Secondary” ASQ factors. Some transcultural differences and possible research approaches are addressed.

## 1. Introduction

Attachment theory has been widely used in understanding relational styles, attitudes, and beliefs in adults. This theoretical framework allows us to understand the influence of past interpersonal history on each member of the current intimate relationship [1]. Therefore, the role played by Bowlby’s model [2] of early bonding of the child can be adapted to their later affective experiences [3,4,5].

The theory of attachment claims the propensity of the human being to create strong affective bonds with specific people [6,7]. John Bowlby was the first one who formulated the basic principles of the theory, giving rise to a new view about the bond created between the child and his mother, as well as the disturbance that the child feels during separation, deprivation, and loss. Mary Ainsworth [8], on the other hand, tested Bowlby’s ideas empirically, helping to expand the theory itself. According to Bowlby’s theory, children internalize the experience they live with their primary caregivers in a way that their early attachment relationships end up forming a pattern of behavior in their future relationships [6] or Internal Working Model [2,9,10,11,12,13,14,15].

In this way, research showed that adults with secure working models manifested better emotional management and experimented with more peer coupling [16]. On the contrary, there is evidence that anxious attachment is significantly more prevalent in psychiatric outpatients and personality disorders than non-clinical participants [17,18,19].

Despite the large number of studies related to attachment, there is no unanimity between researchers about the number of attachment dimensions, both in the way in which they should be classified and in the way they should be structured [20]. Some researchers [21] support the existence of two dimensions of attachment (self/other perception), that generates five-type attachment styles (Confidence, Discomfort with Closeness, Need for Approval, Preoccupation with Relationships, and Relationships as Secondary). Other researchers [6,22] support two dimensions (anxiety/avoidance) that generate four-type attachment styles (Secure, Preoccupied, Fearful, Avoidant). Simultaneously, other researchers [23] support three-type attachment styles (secure, avoidant, and anxious/ambivalent).

The difficulty in defining and clustering attachment styles is due to the differences in how one’s experiences can impact the development of different internal working models, beliefs, and the individual’s way of exploring the environment and the relationships, which also depends on social and cultural factors. On the one hand, some studies consider that the manifestation of one type of attachment style is stable throughout the lifetime and incompatible with others [24]. On the other hand, other empirical studies propose that even if everyone shows a principal attachment style, different attachment styles can manifest in the same person, and can be described most accurately as a dimensional construct [25,26,27]. In any case, the debate is open on whether attachment style is immutable from early childhood or is alterable and subject to change based on experience. Another debate is open on whether attachment style comes from universal patterns of caregiving, and how worthwhile is each pattern on each style according to a specific culture may be focus of attention.

This study focuses on Feeney, Noller and Hanrahan’s five-type attachment model, which converges into the Attachment Style Questionnaire (ASQ) [21]. The ASQ evaluates adult relationships in general and not only in romantic relationships or parental bonding style and is based on Bartholomew and Horowitz’s four-type attachment model [6,28] which is, at the same time, based on Hazan and Shaver’s three-type attachment model [23]. Diagram 1 represents the relationship between the factors of these three progressive models.

In the original English-Australian ASQ Questionnaire [21], five factors explained 43.3% of the variance. These factors were: F1. Confidence (secure attachment), F2. Discomfort with closeness (Hazan and Shaver’s avoidant style), F3. Need for approval (Bartholomew and Horowitz’s fearful and preoccupied styles), F4. Preoccupation with relationships (Hazan and Shaver’s anxious/ambivalent style and Bartholomew and Horowitz’s preoccupied style), F5. Relationships as secondary (Bartholomew and Horowitz’s dismissing style).

The original study of the ASQ questionnaire [21], with an English-Australian sample (N = 470), revealed high internal consistency in all five factors (Table 1). Moreover, the ASQ original study showed correlations with other family-related and personality questionnaires: The Family Functioning Scales (ICPS) [29] and the Eysenck Personality Questionnaire (EPQ) [30].

Regarding the ICPS questionnaire, the perception of high family intimacy, democratic parenting and low levels of family conflict corresponded to high scores in the ASQ F1 factor (Confidence), and low scores in the ASQ F2, F3, F4, and F5 insecure attachment factors.

Regarding the EPQ questionnaire, scores on the neuroticism scale correlated positively with the ASQ F3 and F4 factors (Need for approval and Preoccupation with relationships); on the other hand, the scale of extroversion correlated positively with the ASQ F1 factor (Confidence) and negatively with ASQ F2 and F5 factors.

Currently, there is not yet any publication that validates the ASQ in the Spanish and Japanese populations, while there is an Italian validated ASQ version [20].

The first attempt to translate and validate the ASQ in the Spanish population was between 1999 [31] and 2008 [32]. These authors translated the questionnaire into Spanish and modified the original English-Australian ASQ, giving rise to the Adult Attachment Questionnaire (CAA).

While the original English-Australian ASQ [21] consisted of 40 items and five factors that explained 43.3% of the variance (Confidence; Discomfort with closeness; Need for approval; Preoccupation with relationships; Relationships as secondary), the Spanish CAA consisted of 40 items and four factors that explained 40% of the variance (Low self-esteem, need for approval and fear of rejection; Hostile resolution of conflicts, resentment, and possessiveness; Expression of feelings and comfort with relationships; Emotional self-sufficiency and discomfort with intimacy).

Therefore, this study aimed to validate the Spanish version of the ASQ, comparing it with Italian and Japanese cultures using its original English-Australian five factors structure. Therefore, it was impossible to use the Spanish CAA questionnaire because it was not comparable to other countries.

Based on the aforementioned background surrounding the ASQ, the objectives of this study are to:

1. Translate the original English-Australian ASQ questionnaire to Spanish and explore its validity and reliability in a non-clinical sample of adults.
2. Compare the psychometric properties of the Spanish ASQ questionnaire with two non-clinical samples of Italian and Japanese adults.
3. Compare the psychometric properties of the ASQ in Spanish, Italian, and Japanese samples with the original Australian research [21].

### Hypothesis

**Hypothesis** **1** (H1)**.**  *The ASQ Spanish version applied to a non-clinical sample reflects a five-factor structure like the original ASQ English-Australian study [21].*

**Hypothesis** **2** (H2)**.**  *Considering the ASQ factor loadings of the original English-Australian study, there will be a difference in the weighting of each factor depending on the culture.*

**Hypothesis** **3** (H3)**.**  *The factorial structure of the ASQ in Spanish, Italian, and Japanese non-clinical samples is similar, but with different intensity of patterns depending on the culture.*

## 2. Materials and Methods

### 2.1. Participants

All participants were non-clinical young adults assessed in 2016 and who participated voluntarily (Table 2). A brief sociodemographic and clinic-screening questionnaire was adopted to understand the participants’ education level, and screen for previous psychological or psychiatric treatments to include only non-clinical participants.

Three hundred and fifty-four participants were recruited from university students of health sciences at the European University of Madrid—Spain (36%), University of Trento—Italy (22%), and University of Nagasaki—Japan (34%). One hundred and sixty-nine participants (48%) were male, and one hundred and eighty-five (52%) were female; the mean age of the whole sample was 22.90 years (SD 4.41).

One hundred and thirty-nine Spanish participants (36% of the whole sample), with a mean age of 24.01 (SD 5.46), were divided into fifty-six males (40%) and eighty-three females (60%). Eighty-five Italian participants (22% of the whole sample), with a mean age of 22.76 (SD 3.17), were divided into twenty-six males (31%) and fifty-nine females (69%). One hundred and thirty Japanese participants (34% of the whole sample), with a mean age of 21.79 (SD 3.49), were divided into eighty-seven males (67%) and forty-three females (33%).

Informed consent was sought at each site where data were collected. The current study protocol was approved (CEIm PY:17/20) by the ethical Committee of the Getafe Hospital (Madrid, Spain). The complete dataset to the following Mendeley address: https://figshare.com/s/954da4c6872120ca020d.

### 2.2. Measurements

This study’s instruments consisted of two measures: a brief sociodemographic and clinic-screening questionnaire, and the ASQ questionnaire in Spanish, Italian, and Japanese. Three researchers (O.L.N.; G.I.; C.A.) translated the Attachment Style Questionnaire—ASQ [21] into Spanish independently (see Supplementary Table S1); then, a consensus translation was reached. The translation was checked through back-translation by a native English-speaking professional translator. The same procedure was employed for the Italian and Japanese samples.

The Attachment Style Questionnaire—ASQ [21] consists of 40 items. The ASQ requires the participant to rate aspects of him/herself and others on a Likert scale of 6 points, ranging from (1) “Strongly disagree” to (6) “totally agree” in order to evaluate attachment style in general relationships.

### 2.3. Procedure

Spanish, Italian, and Japanese participants were assessed individually in 2016 at the European University of Madrid (Spain), University of Trento (Italy) and University of Nagasaki (Japan), respectively. Data were collected from one individual face to face sessions of 40 min approximately. In each session, the administration sequence of the questionnaires was the following: 1st. Sociodemographic and clinical survey; 2nd. Attachment Style Questionnaire—ASQ.

### 2.4. Overview of Analysis

For data analysis, descriptive and inferential statistics were carried out. Firstly, we calculated average, standard deviation, skewness, kurtosis, and Cronbach’s α for the five ASQ scales in the Spanish, Italian, and Japanese groups. Then, we explored gender and group differences on the five ASQ factors through one-way MANOVA for independent samples. Age and five ASQ factors correlations were explored using Pearson’s r.

To understand the structure and item-factor loadings of ASQ factors in Spanish, Italian, and Japanese samples, we ran an exploratory factor analysis with oblique rotation (Promax). We opted for an Exploratory Factor Analysis (EFA) since the Confirmatory Factor Analysis (CFA) with the maximum likelihood technique can be affected by inferential measurements by Likert-type items such as those of the ASQ [33,34,35]. Besides, due to the restrictive CFA assumptions, the maximum likelihood confirmatory models may not fit well and has serious problems when used to examine any personality structure [36,37]. According to [38], when the data structures are complex, the CF-Equamax and CF-Facparsim rotations give a similar result and place a greater emphasis on the complexity of the factors, instead of emphasizing the number of items. In particular, the CF-Varimax, CF-Equamax, and CF-Parsimax rotations produce identical solutions if the K value is equal between them. The CF-Varimax rotation with (k: 0.03) emphasizes the complexity of the columns more than a CF-Equamax rotation with (k: 0.05) and a CF-Parsimax (k: 0.07), where K is a parameter that relates to the level of variability and complexity of each factor based on the number of factors and variables. Ref. [39] indicated that if the EFA correlation matrix shows scores equal to or greater than 0.32, it means that if 10% or more of the variance of the factors overlap, an oblique rotation is required (Direct Oblimin or Promax), while if correlations under 0.32 an orthogonal rotation is recommended (Varimax, Quartimax or Equimax). Finally, we calculated an algorithm for the ASQ questionnaire based on the Z scores of each sample (Spanish, Italian and Japanese). The scores of this algorithm were used to compare the ASQ profile in the three cultures.

## 3. Results

Descriptive statistics and internal consistency coefficients (Cronbach’s α) for the ASQ scales in the Spanish, Italian and Japanese samples are shown in Table 3. The skewness and kurtosis values were small for all the scales in the Spanish sample, indicating a mesokurtic distribution. In Italian and Japanese samples, skewness and kurtosis values were small for all the scales except for Japanese F3 (Need for Approval) and F5 (Relationships as Secondary), and Italian F4 (Preoccupation with Relationships), that show a leptokurtic tendency. The internal consistency coefficients were acceptable, considering the number of items constituting each scale.

**Figure 1:**
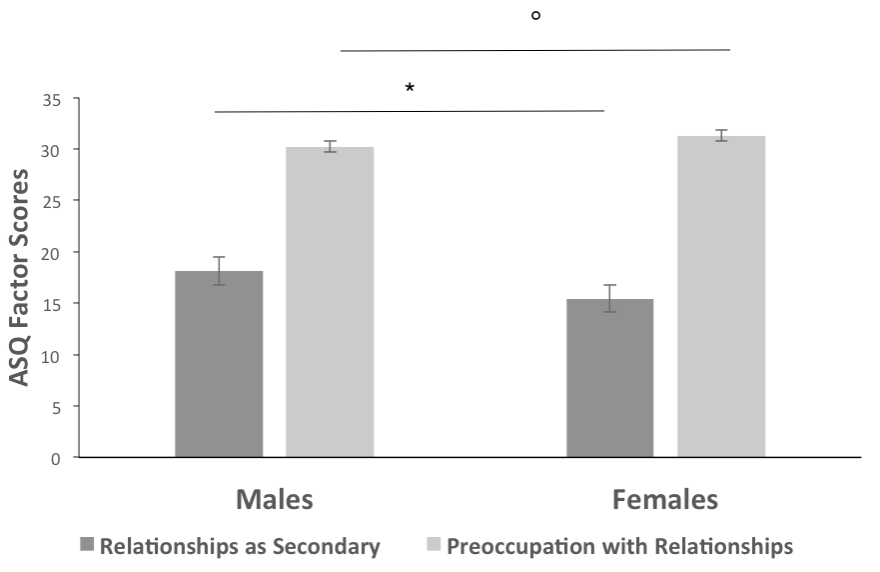
ASQ gender differences in the Italian Sample. Factor score averages. ° *p* < 0.10, * *p* < 0.05.

One-way MANOVA for independent samples (Spanish, Italian and Japanese) on the five ASQ factors shows no gender differences for Spanish (F (5, 133) = 0.793, *p* = 0.56; Wilk’s λ = 0.971, partial $\eta^{2}$ = 0.029) and Japanese groups (F (5, 118) = 0.853, *p* = 0.52; Wilk’s λ = 0.965, partial $\eta^{2}$ = 0.035). On the other hand, for the Italian sample (Figure 1), the results show a significant gender difference (F (5, 79) = 2.757, *p* = 0.05; Wilk’s λ = 0.851, partial $\eta^{2}$ = 0.149). Italian females show lower scores than males on the ASQ factor “Relationships as Secondary” (F (1, 83) = 5.095; *p* < 0.05; partial $\eta^{2}$ = 0.058), and higher scores on the ASQ factor “Preoccupation with Relationships” (F (1, 83) = 3.794; *p* = 0.055; partial $\eta^{2}$ = 0.44).

The results show no age correlation of the ASQ factors for the Japanese group. On the other hand, for the Spanish and Italian samples, there is a positive correlation between age and factor F1 (Confidence), and a negative correlation for the F5 (Relationships as Secondary). Moreover, in the Spanish group, the Factor F2 (Discomfort with Closeness) is also negatively correlated with age (Table 3).

An exploratory factor analysis with oblique rotation (Promax) was applied to Spanish, Italian, and Japanese samples since some correlation values between factors exceeded r: 0.32. The Kaiser-Meyer-Olkin index showed factorial analysis adjusted to the three samples (KMO-Spanish = 0.70, *p* = 0.00; KMO-Italian = 0.69, *p* = 0.00; KMO-Japanese = 0.79, *p* = 0.00). According to [39], an oblique rotation in the Exploratory Factor Analysis is recommended when correlation values between factors are equal to or greater than r: 0.32.

In the Spanish sample, the total variance of the ASQ questionnaire explained for the five factors is 43.67%, similar to the 43.3% obtained in the original English-Australian ASQ study by [21] and other studies, such as that of [20] for the Italian version of ASQ that explained 41.5% of the variance (Table 4). The value of the RMS difference (root mean square error, [40]) between the observed and reproduced correlation matrices was relatively small (0.06), which implies a good fit.

The total variance of the ASQ questionnaire explained in Italian and Japanese samples for the five factors is 49.37% (ITA) and 52.27% (JAP) respectively, superior to the original English-Australian ASQ study (Table 5). In these two cases, the value of the RMS (<0.06) implies a good fit.

Considering the five factors loadings of the original English-Australian ASQ study [21], a one-way MANOVA was run for the three samples (Spanish, Italian and Japanese). The results show significant differences between the three cultures (F (10, 682) = 37.254, *p* < 0.05; Wilk’s λ = 0.418, partial $\eta^{2}$ = 0.353).

**Figure 2:**
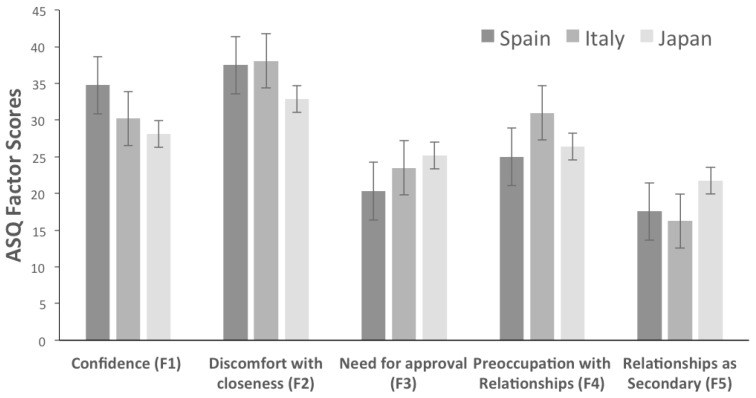
Spanish, Italian and Japanese ASQ factor score averages. In rows, from left to right: (**F1**) ASQ-Confidence. (**F2**) ASQ-Discomfort with Closeness. (**F3**) ASQ-Need for Approval. (**F4**) ASQ-Preoccupation with Relationships. (**F5**) ASQ-Relationships as Secondary.

Tukey’s Post-Hoc shows differences in the all the factors. Specifically, compared with the Japanese group (Figure 2), Mediterranean participants (Spanish and Italian) show higher scores in “Confidence” (F1) and “Discomfort with closeness” (F2), and lower scores in “Need for approval” (F3) and “Relationships as Secondary” (F5) . The Spaniards have higher scores in “Confidence,” and lower scores in “Need for approval” (F3) and “Preoccupation with relationships” (F5). Italians score the highest with the factor “Preoccupation with Relationships” (F4).

An algorithm was applied to the ASQ results, calculating the Z scores of each ASQ factor for each independent sample (Spanish, Italian, and Japanese), using the original English-Australian ASQ five factor loadings [21].

This algorithm (Table 6) allowed the definition of two ASQ main profiles: the balanced profile and unbalanced one. For the balanced profile (secure high), two conditions must be present: (a) the Z score for the confidence factor (F1) had to be higher than −1 SD; (b) the Z score for the other four the factors (F2, F3, F4, and F5) had to be lower than +1 SD. If two conditions were true, the profile was highly balanced and secure (level 9, Table 6). If not true, the profile was classified as unbalanced through one of the additional eight attachment styles, according to the specific profile.

Taking into account that the three groups were non-clinical samples, the results show no significant differences in the algorithm comparisons (Spanish average: 7.69; SD: 2.02; Italian average: 7.54; SD: 2.07; Japanese average: 7.71; SD: 2.05; one-way ANOVA results shows F(2, 351) = 0.58, *p* = 0.56). In all groups, the algorithm average ranges around the ASQ attachment level of “secure low” (level 8) and “avoidant” (level 7).

On the one hand, the exploratory factor analysis, applied to all samples (Table 4), reflected a 5-factor structure like the original English-Australian ASQ study. On the other hand, the current study indicates that some items load in factors differently for each sample (Spanish, Italian, Japanese), that was not found in the original English-Australian study (Table 1).

Considering the new ASQ item-factor loadings independently for Spanish, Italian, and Japanese groups (Supplementary Table S1), a one-way MANOVA was run. The results show significant differences between the three groups (F (10, 694) = 861.41; *p* = 0.000; Wilk’s λ = 0.006, partial $\eta^{2}$ = 0.925). These differences (Table 6) are the same as those found using the original English-Australian ASQ factor loadings, with the addition of a difference between Spaniards and Italians for factors F2 (“Discomfort with closeness”) and F3 (“Need for Approval”), and between Spaniards and Japanese for factor F5 (“Relationships as Secondary”).

**Figure 3:**
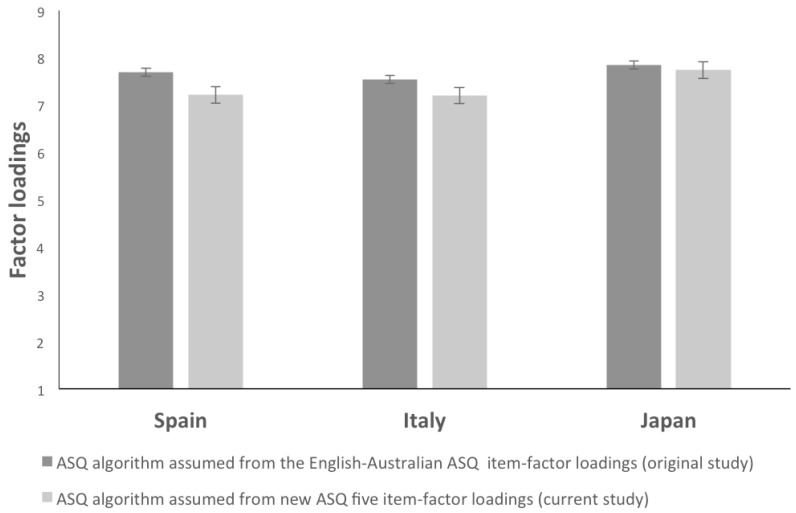
ASQ algorithm average in Spanish, Italian, and Japanese groups using the original English-Australian factor loadings and the five factor loadings (factorial exploratory analysis) of the current study.

As in the case of the ASQ algorithm (Supplementary Table S1) assumed from the original English-Australian ASQ five factor loadings [21], also taking into account the ASQ algorithm assumed from new item-factor loadings (actual study, Figure 3), no significant differences were detected between groups (Spanish mean: 7.22; SD: 2.06; Italian mean: 7.20; SD: 2.05; and Japanese mean: 7.74; SD: 1.89; one-way ANOVA results shows F(2, 351) = 2.887, *p* = 0.057). In both cases (the original ASQ study and our study) the ASQ style algorithm average ranges around level 8 (secure low), and 7 (avoidant).

## 4. Discussion

This study aimed to translate and explore the Spanish version of the Attachment Style Questionnaire (ASQ), comparing it with Italian and Japanese translations and the original English Australian’s validated version [21].

First, we translated the ASQ questionnaire to Spanish, Italian, and Japanese, checking it through back-translation and exploring their consistency and factorial structure in a non-clinical sample of adults. We found that the internal consistency coefficients for the Spanish, Italian, and Japanese ASQ were acceptable for all the countries.

Then we compared ASQ Spanish psychometric properties with two non-clinical samples of Italian and Japanese adults. No gender differences for all ASQ factors in the Spanish and Japanese groups were found. In contrast, in the Italian group, females showed lower scores in “Relationships as Secondary” and higher scores in “Preoccupation with Relationships”. On the other hand, concerning Attachment security and the “Confidence” factor, Italian females showed levels similar to the general Italian population [20], the Australian female population [41], and equivalent to the general Swedish population [42], but lower than the pregnant women in Australia [43] and Sweden [44].

There is a proportion of parenting that is culturally based [45]. Parents in different cultures perform different ways of raising their babies [46]. However, there are common patterns related to how children need to be nurtured and protected to ensure the survival of the children that overcome cultural standards and values [47]. Attachment theory, according to [11], claims that there are genetically predisposed patterns of behavior developed by significant others that allow the biological adaptability of the offspring and, in turn, the preservation of the species. Moreover, attachment theory also states that bonding is not exclusive to mothers but rather to any person considered ready enough to provide a secure base that supports and fosters the child’s discovery of the environment [2]. This perspective follows from contemporary ethology and the evolution theory [7] which states that attachment involves fathers, older siblings, or grandparents and is not just focused on the child-mother dyad. Therefore, even though there are differences between Western European and Eastern Asian family structures, where nonmaternal caregivers are also important bonding figures, any personal network of relationships has performed attachments with almost the same mechanism suitable for providing protection and security [47].

According to ecocultural theory [48], the environment and parental practices are equally important for the child’s development. For example, Eastern Europeans show more preoccupied attachment than Western Europeans [49]. This difference is mainly due to different parenting practices than to the direct influence of the culture on an individual [50]. Regarding Italian culture, the fact that women express more preoccupation with relationships may depend on a specific Italian cultural aspect. Different studies found specific cultural variations in the concept of motherhood and female identity in Italy. There is evidence that Italian women’s upbringing models further foster interdependence with offspring and likely is relevant to interpersonal relationships [51,52].

No age correlation for all ASQ factors in the Japanese sample was found. At the same time, the Mediterranean cultures (Spanish and Italian) show a positive correlation with “Confidence” and a negative correlation with “Relationships as Secondary” and age. Moreover, in the Spanish sample, age correlates negatively with “Discomfort with Closeness” too.

The fact that the Japanese Attachment pattern stays steady throughout the lifetime may be because parent-child dynamics in Eastern Asian cultures remain stable throughout life. A study about parent-child attachment compared the cycle and development of close relationships between the USA and Japan [53]. The authors suggest that the shift of attachment from parents to peers, typical from adolescence and adulthood, leads to a more independent Western culture relationship. However, in Eastern Asiatic cultures, the parent-child relationship is characterized by maintaining interdependence, based on children’s obedience, creating a debt where the children are compelled to honor and be devoted towards parents’ wishes for the rest of their lives. These differences follow developmental psychology theories that state the existence of two distinct developmental pathways: the route of independence and interdependence [54]. As peers and romantic relationships depend on the evolution of the relationships with the family of origin, it is understandable to maintain attachment patterns in Eastern societies.

From this perspective, Western-Mediterranean cultures promote more openness from a perspective of individual independence, which varies in a lifetime and can explain the shift towards greater confidence and less perception of relationships as secondary (and less discomfort with them in Spanish culture), differently from Eastern-Japanese culture. In the Western-Mediterranean culture, the evolution of attachment pattern moves from the family to new relationships outside the nucleus of birth, without abandoning the family of origin. The Japanese attachment pattern, more stable over time, reflects a more significant promotion of the individual’s interdependence with more stable attachment values during a lifetime.

We also compared the ASQ psychometric properties in Spanish, Italian, and Japanese samples with the original English-Australian ASQ study [21]. Regarding Hypothesis 1 (according to which posited that the ASQ Spanish version reflects a 5-factor structure like the original Australian study) results showed that a 5-factor structure explains the 43.67% of the variance in the Spanish ASQ version, and respectively the 49.37% for the Italian and 52.27% for the Japanese ASQ versions. These results are similar to the original study, implying a good fit.

The results of previous validation studies of the ASQ in Spanish [31,32] preferred to settle for a 4-factor structure. This Spanish study explained 40% of the variability, transforming the original ASQ into a different questionnaire, suitable only for Spanish culture and not allowing any cultural comparison. Our study found that a 5-factor structure explains 43.67% of the variance for the Spanish ASQ, like the original English-Australian ASQ study, allowing comparison between countries. Indeed, the 5th factor of the ASQ “Relationship as Secondary” could be operationalized with more efficient items that could increase this dimension’s explained variability (3.67% of variability more than a 4-factor structure). Going deeper into this 5th factor could be the object of further ASQ revision studies.

As for Hypothesis 2, the results confirm differences in attachment style between Spanish, Italian, and Japanese cultures. In factor F1, Spaniards are more “confident” than Italians, and Italians are more “confident” than Japanese. Regarding factor F2, Mediterranean samples (Spanish and Italian) score higher in “Discomfort with Closeness” than Japanese. In factor F3, Japanese participants show higher levels of “Need for Approval” than Mediterranean cultures. In the factor F4, Italians show higher “Preoccupation with Relationships” than Japanese and Japanese than Spaniards. Finally, in the factor F5, Japanese show higher levels of “Relationships as Secondary” than Mediterranean cultures. In other words, compared to Japanese participants, the Western European ones are more confident (F1), show more discomfort with closeness (F2), less need for approval (F3) and consider the relationships as secondary (F5) less. Spaniards are the most confident (F1), those who need the least for approval (F3), and the least preoccupied with relationships (F5). Italians are the most preoccupied with relationships (F4), including more than the Spaniards, like the previously cited study between Eastern and Western Europeans [49]. Western European societies like Spain and Italy are more individualistic than Eastern Asian societies. For example, in an Eastern Asian culture like China, people live in a collective culture where each person evaluate him/herself in terms of interconnectedness and the value they provide to others, more than people from Western European cultures [50,55,56]. Moreover, insecure romantic attachments are associated with the harsh environment and economic hardships [57], and preoccupied attachment co-occurred with high rates of collectivism [58].

The fact that Eastern Japanese culture needs more approval from the other person and greater perception of preoccupation with relationships comes from Ancient Samurai´s Honor beliefs and philosophers’ values, currently applicable in that culture, such as Confucian doctrine related to loyalty and the supremacy of the group over the individual [59]. This forced collectivism is in high contrast with the individualism fostered in Western cultures [60], where autonomy is crucial for developing the self. However, in Western cultures, the attachment is increasingly focused on non-parental figures and peers. Therefore, intimate relationships become extremely important in adulthood [61]. The maintenance of these relationships must be ensured with emotional contact based on trust, confidence, and prosocial attitudes, such as self-regulation and actions that contribute to the partner [47]. In this regard, our results follow the “Investment model” [62], according to which there is a necessity to develop strong “confidence” and trust with the new sources of trust (the peers) in order to build up a strong bond in intimate relationships. At the same time, higher levels of self-regulation should be shown, probably to avoid the dissatisfaction and abandonment of the partner, which may lead towards some fear-based avoidant-anxious manner or “discomfort with closeness” in the subject [63].

Regarding the factor F5, Italians (particularly Italian women) agree less strongly with “relationships as secondary” than Spanish and Japanese.

These results follow [64], where Italian female adolescents reported a stronger attachment to their parents than males. Understandably, this internal working model of attachment transferred from parents to peers remains steady over time [61], which could have explained why Italian women follow a more “preoccupied with relationships” style. In this line, another study found that Italian wives were less satisfied than husbands [65], and this could come from the fact that this relationship matters more to female than males. Women could have higher expectations, dedication, and commitment while receiving a different attitude from males.

Regarding the Hypothesis 3 (ASQ factorial structure of the ASQ in Spanish, Italian and Japanese versions show similar patterns but with different intensity), results indicate that some items load in factors other from those of the original English-Australian study. The results show more remarkable differences between the three cultures when using the new item-factor loadings (following the current research). Regardless, considering each sample’s features, if we calculate an ASQ algorithm, using both the original item-factor loadings, both the new item-factor loadings, no significant differences are detected. Considering that the three groups are non-clinical samples, both the original item-factor loadings, both the new item-factor loadings, Spanish, Italian, and Japanese participants, show a global ASQ attachment style around secure low (level 8) and avoidant (level 7).

It means that the algorithm calculation proposal, suggested in this article, could constitute a method towards an evolution of attachment style classifications.

### 4.1. Limitations

This study found that the 5th factor of the ASQ questionnaire “Relationship as Secondary” in Spanish, Italian, and Japanese translations can be operationalized with more efficient items that can increase this dimension, explaining more variability. Going deeper into this 5th factor could be the object of further ASQ revision studies.

The Italian sample shows a small sample size (less than 100) and a gender unbalance (26 males and 59 females) compared with other groups. Moreover, ASQ factor 4 (Preoccupation with Relationships) shows a leptokurtic tendency in the Italian sample. These characteristics could have influenced the findings related to gender differences in the Italian sample for factors F4 (Preoccupation with Relationships) and F5 (Relationships as Secondary).

Although the sociodemographic questionnaire allowed a screening measure of the non-clinical sample (eliminating false positives and undiagnosed clinical population), a clinical evaluation and other attachment questionnaires would have added reliability to the research.

Another limitation of the study is not determining the possible existence of the underlying dimensions of anxiety and avoidance [41] in the ASQ analysis, using the original item-factor loadings and the current item-factor loadings through the algorithm calculation. Finally, the study does not explore the Spanish, Italian, and Japanese ASQ external validity. It could be interesting to explore the external validity with another attachment questionnaire like the Experience in Close Relationships Scale—ECR [27]. A future study should be developed with this aim, comparing these Spanish, Italian, and Japanese versions of the ASQ questionnaire with the validated versions of the ECR scale in Spanish [66], Italian [67], and Japanese [68].

### 4.2. Implications

This study’s research findings increase the knowledge of the attachment perceptions of non-clinical adults in different cultures. Western Mediterranean cultures (Spain and Italy) foster a more flexible and independent bond throughout life. Eastern-Asian culture (specifically Japan) fosters more stable attachment patterns between parents (according to the grown-up children).

The algorithm suggested in this article could constitute a method towards an evolution of attachment style classifications. It can allow comparisons between more classical classifications (Bowlby and Ainsworth: Safe, Insecure-Avoidant, Insecure-Ambivalent, Insecure-Disorganized) with conceptual attachment evolutions (such as those of Bartholomew and Horowitz: Secure, Dismissing, Fearful and Preoccupied; and Hazan and Shaver: Secure, Avoidant, and Anxious/Ambivalent).

Considering that attachment theory is a broad field of study that encompasses parents’ relationships, peers, and romantic relationships, it is plausible to find an effect of culture in the attachment patterns in intimate relationships.

This study’s research findings claim that cultural aspects influence attachment. Probably in Western Mediterranean cultures, adults show more flexible and more changeable attachment towards their relationships than Eastern Japanese culture.

Although the Mediterranean cultures increase individuals’ autonomy, individuality, and independence, interpersonal relationships are a source of psychological well-being and emotional stability.

Interpersonal confidence is a basic necessity that needs to be satisfied, as infants require parents’ loving care and attention. Insecure attachment styles can generate psychopathological conditions and feelings of loneliness, giving place to the expression of a biological need that compels humans toward seeking emotional connections in order to avoid the feeling of disengagement [69].

Not only cultural aspects are essential in the study of attachment. Unwanted and traumatic events that a person can experience can characterize new relationships or generate difficulties, discomfort, and psychopathologies [70,71].

Marital separation and divorce are the most stressful life events, only surpassed by the death of a loved person [72,73]. These events’ implications can have serious health consequences that need psychological therapies that understand the attachment processes [63,74] and the influence of environmental factors such as culture or the psychotherapeutic relationship.

## Figures and Tables

**Table 1:** Within group correlations and Cronbach’s alphas of the original ASQ dimensions, Australian study (N = 470).

	Items	F1		F2		F3		F4		F5	
		α	***r***	α	***r***	α	***r***	α	***r***	α	***r***
F1	1,2,3,19,31,37,38 (rev. 33)	0.80	-		−0.52		−0.39		−0.33		−0.18
F2	4,5,16,17,23,25,26,34 (rev. 20, 21)			0.84			0.31		0.31		0.44
F3	11,12,13,15,24,27,35					0.79			0.57		0.16
F4	18,22,28,29,30,32,39,40							0.76	-		0.17
F5	6,7,8,9,10,14,36									0.76	-

F1 = Confidence; F2 = Discomfort with Closeness; F3 = Need for Approval; F4 = Preoccupation with Relationships; F5 = Relationships as Secondary; α = Cronbach’s α; *r* = Pearson’s *r* (*p* < 0.01); rev = reversed item. From Feeney, Noller and Hanrahan, 1994, pp. 136–139.

**Table 2:** Participants.

	Age				Sex		
	Min	Max	Average	SD	Female	Male	Total
Spain	18	37	24.01	5.46	83 (60%)	56 (40%)	139
Italy	18	34	22.76	3.17	59 (69%)	26 (31%)	85
Japan	18	37	21.79	3.49	43 (33%)	87 (67%)	130
Total	18	37	22.90	4.41	185 (52%)	169 (48%)	354

**Table 3:** Attachment Style Questionnaire (ASQ) descriptive statistics.

ASQ Factor	Group	M	SD	Skewness	Kurtosis	α
F1	Spain	34.74	5.41	−0.28	−0.11	0.67
	Italy	30.22	5.76	0.02	0.29	0.77
	Japan	28.11	6.81	−0.29	0.61	0.83
F2	Spain	37.48	8.56	−0.24	0.25	0.81
	Italy	38.05	7.27	0.23	−0.46	0.75
	Japan	32.88	8.09	0.33	0.44	0.82
F3	Spain	20.31	6.34	0.25	−0.38	0.73
	Italy	23.47	6.17	0.35	−0.40	0.72
	Japan	25.16	5.22	−0.25	1.36	0.66
F4	Spain	25.00	6.07	0.17	−0.49	0.62
	Italy	30.96	6.18	−0.30	1.20	0.71
	Japan	26.35	6.10	0.10	0.03	0.74
F5	Spain	17.55	5.58	0.43	0.02	0.65
	Italy	16.26	5.16	0.87	0.44	0.76
	Japan	21.77	4.98	0.16	1.11	0.63

Number of items for: F1 (Confidence) = 8; F2 (Discomfort with Closeness) = 10; F3 (Need for Approval) = 7; F4 (Preoccupation with Relationships) = 8; F5 (Relationships as Secondary) = 7; *α* = Cronbach’s *α*.

**Table 4:** Total variance explained of the ASQ factors.

Factor	Sample	IEV Total	IEV Var%	IEV Cum%	Rotation
F1	Spain	7.00	17.51	17.51	5.08
	Italy	7.60	18.99	18.99	6.91
	Japan	10.12	25.30	25.30	8.38
F2	Spain	4.19	10.47	27.98	4.75
	Italy	5.04	12.61	31.60	4.43
	Japan	3.85	9.62	34.92	7.01
F3	Spain	2.41	6.03	34.01	3.11
	Italy	2.79	6.97	38.58	4.04
	Japan	3.03	7.57	42.49	5.77
F4	Spain	2.16	5.40	39.41	3.31
	Italy	2.41	6.02	44.59	4.74
	Japan	2.02	5.05	47.55	3.35
F5	Spain	1.71	4.27	43.68	3.04
	Italy	1.92	4.79	49.38	3.34
	Japan	1.89	4.73	52.28	2.29

F1 = Confidence; F2 = Discomfort with Closeness; F3 = Need for Approval; F4 = Preoccupation with Relationships; F5 = Relationships as Secondary; IEV = Initial Eigen Values; IEV Var = % of Variance; IEV Cum = % Cumulative; Rotation = Rotation Sums of Squared Loadings

**Table 5:** Correlations between ASQ factors and age.

Group	F1	F2	F3	F4	F5
Spain	0.17 *	−0.27 **	−0.02	−0.08	−0.18 *
Italy	0.21 *	−0.17	−0.04	−0.11	−0.22 *
Japan	0.05	0.01	0.12	−0.15	−0.10

F1 = Confidence; F2 = Discomfort with Closeness; F3 = Need for Approval; F4 = Preoccupation with Relationships; F5 = Relationships as Secondary; * *p* < 0.05; ** *p* < 0.001.

**Table 6:** ASQ algorithms.

Attachment Style	Level	ASQ Attachment Style	Algorithm
Secure High	9	Secure High	F1 > −1 SD & F2 < +1 SD, F3 < +1 SD, F4 < +1 SD, F5 < +1 SD
Secure Low	8	Dismissing Low	F1 > −∞ SD, F2 * > +1 SD, & F3 < +1 SD, F4 < +1 SD, F5 < +1 SD
Avoidant	7	Dismissing High	F1 * > −∞ SD, F2 * > +1 SD, F3 > +1 SD & F4 < +1 SD, F5 < +1 SD
Anxious/Ambivalent Low	6	Preoccupied	F1 * > −∞ SD, F2 * > +1 SD, F5 > +1 SD & F3 < +1 SD, F4 < +1 SD
Anxious/Ambivalent Low	5	Fearful	F1 * > −∞ SD, F2 * > +1 SD, F4 > +1 SD & F3 < +1 SD, F5 < +1 SD
Avoidant/Ambivalent Low	4	Dismissing & Preoccupied	F1 * > −∞ SD, F2 * > +1 SD, F3 > +1 SD, F4 * > +1 SD, F5 > +1 SD
Avoidant/Ambivalent High	3	Dismissing & Fearful	F1 * > −∞ SD, F2 * > +1 SD, F3 > +1 SD, F4 > +1 SD, & F5 < +1 SD
Anxious/Ambivalent High	2	Preoccupied & Fearful	F1 * > −∞ SD, F4 > +1 SD, F5 > +1 SD, F2 * > +1 SD & F3 < +1 SD
Disorganized	1	Dismissing, Preoccupied & Fearful	F1 * > −∞ SD, F2 * > +1 SD, F3 > +1 SD, F4 > +1 SD, F5 > +1 SD

Z Scores of: F1 = Confidence; F2 = Discomfort with Closeness; F3 = Need for Approval; F4 = Preoccupation with Relationships; F5 = Relationships as Secondary; * May be present.

## Data Availability

The dataset is released under the Creative Common 4.0 (CC-BY) License at https://figshare.com/s/954da4c6872120ca020d.

## Supplementary Materials

The following are available online at https://www.mdpi.com/2254-9625/11/1/10/s1, Table S1: ASQ item-factor loadings (Original English-Australian vs. Current study) ASQ item multiple-group component loadings in Spanish, Italian and Japanese samples.
