# Supplementary material for: Exploration of the Spanish Version of the Attachment Style Questionnaire: A Comparative Study between Spanish, Italian, and Japanese Culture
Source: Eur J Investig Health Psychol Educ. 2021 Feb 4;11(1):113–28. doi: 10.3390/ejihpe11010010 (PMC8314336; doi:10.3390/ejihpe11010010)
Supplement: Supplementary file 1 [file ejihpe-11-00010-s001.pdf]

Table 1: ASQ item-factor loadings (Original English-Australian vs. Current study) ASQ item multiple-group component loadings in Spanish, Italian and Japanese samples. (1) Original Factor English-Australian ASQ 5 factors loadings (Feeney, Noller, & Hanrahan, 1994): Confidence: F1. (C); Discomfort with Closeness: F.2 (DC); Need for Approval: F.3 (NA); Preoccupation with Relationships: F.4 (PR); Relationships as Secondary: F.5 (RS). (2) Spanish Factor, Actual study, ASQ 5 factors loadings, Spanish Sample: F1. (C); Discomfort with Closeness: F.2 (DC); Need for Approval: F.3 (NA); Preoccupation with Relationships: F.4 (PR); Relationships as Secondary: F.5 (RS). R: Original rotated item; S: Spanish rotated item; I: Italian rotated item; J: Japanese rotated item

**Item**	**O. F. (1)**	**S. F. (2)**	**Original item**	**Spanish item**	**F.1**			**F.2**			**F.3**			**F.4**			**F.5**		
					**Spa**	**Ita**	**Jap**	**Spa**	**Ita**	**Jap**	**Spa**	**Ita**	**Jap**	**Spa**	**Ita**	**Jap**	**Spa**	**Ita**	**Jap**
1	F.1 (C)	F.1 (C)	Overall, I am a worthwhile person.	En general, soy una persona que merece la pena.	0.30	-0.03	0.52	-0.27	-0.11	0.31	-0.11	-0.43	0.10	-0.17	0.44	-0.47	-0.03	0.25	-0.02
2	F.1 (C)	F.4 (PR)	I am easier to get to know than most people.	Soy más fácil de conocer que la mayoría de la gente.	0.25	-0.01	0.53	-0.48	0.40	-0.05	0.25	-0.33	0.29	0.34	0.27	-0.43	0.18	-0.17	-0.18
3	F.1 (C)	F.1 (C)	I feel confident that other people will be there for me when I need them.	Estoy seguro de que otras personas estarán ahí para mi cuando las necesite.	0.32	0.38	0.32	-0.35	0.08	0.16	0.08	-0.56	-0.07	-0.12	0.15	-0.58	-0.25	-0.06	0.04
4	F.2 (DC)	F.5 (RS)	I prefer to depend on myself rather than other people.	Prefiero depender de mí mismo antes que de otras personas.	-0.06	-0.25	0.08	0.44	-0.14	0.58	-0.42	0.19	0.30	-0.01	0.18	0.36	0.22	0.58	0.20
5	F.2 (DC)	F.2 (DC)	I prefer to keep to myself.	Prefiero guardarme mis cosas para mí.	0.37	0.06	0.00	0.36	-0.21	0.26	0.05	0.49	0.04	0.16	-0.14	0.46	-0.26	0.49	0.32
6	F.5 (RS)	F.1 (C)	To ask for help is to admit that you're a failure.	Pedir ayuda es reconocer que has fallado.	0.52	-0.23	0.32	0.20	0.11	0.22	-0.13	0.31	0.14	0.34	0.27	0.48	0.23	0.39	0.00
7	F.5 (RS)	F.1 (C)	People's worth should be judged by what they achieve.	La valía de las personas debería medirse por sus logros.	0.46	-0.46	0.34	0.28	0.29	0.29	-0.36	0.14	0.25	0.29	0.25	0.14	-0.09	0.39	0.33
8	F.5 (RS)	F.1 (C)	Achieving things is more important than building relationships.	Alcanzar mis metas es más importante que construir relaciones.	0.14	-0.15	0.34	0.22	0.31	0.34	-0.34	0.21	-0.08	0.45	0.06	0.14	-0.13	0.64	0.40
9	F.5 (RS)	F.4 (PR)	Doing your best is more important than getting on with others.	Hacerlo lo mejor posible es más importante que estar integrado en el grupo.	0.36	0.01	0.27	0.23	0.33	0.22	-0.42	0.14	-0.16	0.48	0.44	0.25	-0.10	0.52	0.55
10	F.5 (RS)	F.3 (NA)	If you've got a job to do, you should do it no matter who gets hurt.	Si tienes un trabajo que hacer, deberías hacerlo sin importar si eso hace daño a otros.	0.32	-0.15	0.49	0.10	0.47	0.25	0.51	0.19	-0.37	0.14	0.22	0.14	0.24	0.43	0.34
11	F.3 (NA)	F.3 (NA)	It's important to me that others like me.	Gustarle a los demás es importante para mí.	0.02	-0.28	0.17	0.11	0.07	-0.38	0.53	-0.05	0.45	0.14	0.29	-0.13	0.25	-0.55	0.35
12	F.3 (NA)	F.3 (NA)	It's important to me to avoid doing things that others won't like.	Es importante para mí evitar hacer cosas que no les gusten a las otras personas.	0.06	-0.31	0.33	0.23	0.00	-0.27	0.50	0.19	-0.20	0.31	0.16	0.18	0.11	-0.42	-0.07
13	F.3 (NA)	F.2 (DC)	I find it hard to make a decision unless I know what other people think.	Me resulta difícil tomar una decisión sin saber lo que opinan los demás.	0.07	-0.18	0.32	0.22	0.16	-0.49	0.01	0.23	0.01	0.11	-0.30	0.14	0.01	-0.38	0.01
14	F.5 (RS)	F.2 (DC)	My relationships with others are generally superficial.	Mis relaciones generalmente son superficiales.	-0.22	0.12	0.10	0.48	0.04	0.07	0.48	0.39	-0.19	-0.08	-0.12	0.67	-0.07	0.23	-0.01
15	F.3 (NA)	F.2 (DC)	Sometimes I think I am no good at all.	En ocasiones pienso que no valgo para nada.	0.05	0.11	-0.13	0.61	0.10	-0.27	-0.38	0.65	0.00	-0.34	-0.15	0.66	0.08	-0.34	0.19
16	F.2 (DC)	F.2 (DC)	I find it hard to trust other people.	Me resulta difícil confiar en otras personas.	-0.06	-0.12	0.15	0.26	-0.36	0.28	-0.52	0.54	0.07	-0.18	0.17	0.64	0.26	0.14	-0.02
17	F.2 (DC)	F.2 (DC)	I find it difficult to depend on others.	Me resulta difícil depender de otras personas.	-0.03	0.30	-0.08	0.56	-0.46	0.45	0.16	0.09	0.18	-0.14	0.39	0.64	-0.21	0.35	-0.13
18	F.4 (PR)	F.5 (RS)	I find that others are reluctant to get as close as I would like.	Me parece que los demás son reacios a acercarse a mi tanto como me gustaría.	0.17	0.09	-0.02	-0.58	0.37	-0.03	-0.10	0.42	0.12	0.09	0.04	0.74	0.32	-0.03	0.14
19	F.1 (C)	F.4 (PR)	I find it relatively easy to get close to other people.	Me resulta relativamente fácil acercarme a otras personas.	-0.05	-0.12	0.56	-0.56	0.10	-0.13	0.27	-0.56	0.33	0.42	0.32	-0.47	0.12	-0.17	-0.14
20 (R, I)	F.2 (DC)	F.2 (DC)	I find it easy to trust others.	Me resulta fácil confiar en otras personas.	0.03	-0.36	-0.30	-0.28	-0.41	0.44	0.52	0.34	0.04	0.14	0.07	0.44	-0.43	0.30	-0.13
21 (R, I)	F.2 (DC)	F.1 (C)	I feel comfortable depending on other people.	Me siento bien dependiendo de otras personas.	0.40	0.06	-0.27	0.24	-0.68	0.55	0.34	-0.06	0.31	-0.07	0.03	0.44	0.35	0.26	-0.12
22	F.4 (PR)	F.1 (C)	I worry that others won't care about me as much as I care about them.	Me preocupa que los demás no se preocupen de mí tanto como yo me preocupo de ellos.	-0.06	0.01	0.24	0.56	0.04	-0.22	-0.40	0.57	0.21	0.03	0.33	0.60	-0.03	-0.25	0.00
23	F.2 (DC)	F.2 (DC)	I worry about people getting too close.	Me preocupa que la gente se acerque demasiado.	-0.01	0.07	0.17	0.50	0.07	0.06	0.36	0.34	0.19	0.03	0.22	0.55	0.09	0.39	-0.33
24	F.3 (NA)	F.2 (DC)	I worry that I won't measure up to other people.	Me preocupa no estar a la altura de las otras personas.	0.15	0.06	0.14	0.56	0.19	-0.29	-0.24	0.57	0.17	-0.15	0.00	0.72	-0.19	-0.38	0.00
25	F.2 (DC)	F.2 (DC)	I have mixed feelings about being close to others.	Tengo sentimientos encontrados sobre la cercanía con otras personas.	0.04	0.31	0.36	0.71	0.10	0.03	0.09	0.65	-0.12	-0.12	0.19	0.61	-0.25	-0.12	-0.22
26	F.2 (DC)	F.2 (DC)	While I want to get close to others, I feel uneasy about it.	Aunque me gustaría sentirme más cerca de otras personas, no me resulta fácil hacerlo.	0.09	0.30	0.13	0.64	0.03	-0.08	0.21	0.72	0.04	-0.08	-0.01	0.69	-0.13	-0.13	-0.23
27	F.3 (NA)	F.1 (C)	I wonder why people would want to be involved with me.	Me pregunto por qué la gente podría querer relacionarse conmigo.	0.39	0.05	0.29	0.00	0.17	-0.15	0.32	0.67	-0.21	-0.32	-0.13	0.65	0.06	-0.21	-0.03
28	F.4 (PR)	F.3 (NA)	It's very important to me to have a close relationship.	Es muy importante para mí el tener una relación muy cercana.	0.14	-0.09	0.17	-0.24	0.10	-0.35	0.35	0.05	0.37	-0.37	0.42	-0.36	0.23	-0.55	0.45
29	F.4 (PR)	F.3 (NA)	I worry a lot about my relationships.	Me preocupo mucho por mis relaciones.	-0.05	-0.06	0.03	-0.05	-0.13	-0.32	0.50	-0.13	0.09	0.04	0.42	0.75	-0.10	-0.69	0.08
30 (S)	F.4 (PR)	F.2 (DC)	I wonder how I would cope without someone to love me.	No sé cómo me las arreglaría sin alguien que me amase.	0.12	-0.31	0.06	-0.47	0.03	-0.01	-0.09	0.13	0.34	-0.23	0.33	0.59	0.03	-0.51	-0.10
31 (I)	F.1 (C)	F.2 (DC)	I feel confident about relating to others.	Me siento confiado/a en mi relación con otras personas.	-0.06	0.16	0.51	0.51	0.06	0.18	0.43	-0.62	0.30	0.00	0.46	-0.52	0.08	0.03	-0.19
32	F.4 (PR)	F.2 (DC)	I often feel left out or alone.	Suelo sentirme abandonado/a o solo/a.	-0.08	0.07	0.01	0.63	-0.08	-0.21	0.35	0.63	-0.17	-0.08	0.19	0.67	-0.06	-0.31	0.23
33 (R)	F.1 (C)	F.2 (DC)	I often worry that I do not really fit in with other people.	Suele preocuparme el creer que realmente no encajo con otras personas.	-0.12	-0.23	-0.05	0.48	0.05	0.45	-0.01	-0.68	-0.04	0.19	-0.02	-0.51	0.24	0.38	-0.16
34	F.2 (DC)	F.2 (DC)	Other people have their own problems, so I don't bother them with mine.	Las otras personas tienen sus propios problemas y preocupaciones, así que no las molesto con los míos.	-0.05	0.31	-0.18	0.60	0.09	0.01	0.19	0.53	0.36	0.29	0.18	0.42	-0.09	0.11	0.08
35	F.3 (NA)	F.2 (DC)	When I talk over my problems with others, I generally feel ashamed or foolish.	Cuando hablo con otras personas sobre mis problemas, generalmente me siento avergonzado/a o tonto/a.	0.09	0.17	-0.10	0.37	-0.09	-0.22	-0.26	0.55	0.22	0.29	0.14	0.46	-0.07	-0.05	-0.13
36	F.5 (RS)	F.2 (DC)	I am too busy with other activities to put much time into relationships.	Estoy muy ocupado/a con otras actividades como para dedicar mucho tiempo a las relaciones personales.	-0.12	0.10	0.40	0.29	0.31	-0.16	-0.37	0.46	0.10	-0.09	0.05	0.34	0.55	0.54	-0.36
37	F.1 (C)	F.1 (C)	If something is bothering me, others are generally aware and concerned.	Cuando algo me molesta, los demás generalmente son conscientes de ello y se preocupan.	0.42	0.42	0.23	-0.15	0.13	-0.20	-0.17	-0.33	0.00	-0.28	0.26	-0.39	-0.30	0.03	0.10
38	F.1 (C)	F.1 (C)	I am confident that other people will like and respect me.	Estoy seguro/a de que les gustaré a otras personas y que me respetarán.	0.52	0.45	0.58	-0.37	0.12	0.16	-0.13	-0.54	0.06	-0.21	0.34	-0.51	-0.06	0.12	-0.09
39	F.4 (PR)	F.1 (C)	I get frustrated when others are not available when I need them.	Me frustra que otras personas no estén disponibles cuando las necesito.	0.49	-0.01	0.56	0.25	-0.39	0.03	0.20	0.19	-0.40	-0.31	0.46	0.37	0.09	-0.21	-0.05
40	F.4 (PR)	F.2 (DC)	Other people often disappoint me.	Las otras personas me decepcionan a menudo.	0.25	-0.10	0.46	0.61	-0.23	0.07	0.20	0.52	-0.37	-0.17	0.41	0.49	0.22	0.09	-0.23

ASQ item-factor loadings (Original English-Australian vs. Current study) ASQ item multiple-group component loadings in Spanish, Italian and Japanese samples. (1) Original English-Australian ASQ 5 factors loadings (Feeney, Noller, & Hanrahan, 1994). (2) Current study, ASQ 5 factors loadings. F1 Confidence (C); F2 Discomfort with Closeness (DC); F3 Need for Approval (NA); F4 Preoccupation with Relationships (PR); F5 Relationships as Secondary.
F1. (C); Discomfort with Closeness: F2 (DC); Need for Approval: F3 (NA); Preoccupation with Relationships: F4 (PR); Relationships as Secondary: F.5 (RS).
R: Original rotated item; S: Spanish rotated item; I: Italian rotated item; J: Japanese rotated item
